# Draft Genome Sequence of the *Serratia marcescens* Strain VGH107, a Taiwanese Clinical Isolate

**DOI:** 10.1128/genomeA.00249-13

**Published:** 2013-05-16

**Authors:** Po-Yu Liu, Yao-Ting Huang, Sheng-Yi Lin, Gee-Cheng Chang, Jian-Wei Chen

**Affiliations:** Department of Internal Medicine, Taichung Veterans General Hospital, Taichung, Taiwana;; Department of Computer Science and Information Engineering, National Chung Cheng University, Minxiong, Taiwanb;; Institute of Biomedical Sciences, National Chung Hsing University, Taichung, Taiwanc;; Agricultural Biotechnology Center, National Chung Hsing University, Taichung, Taiwand

## Abstract

*Serratia marcescens*, a member of the family *Enterobacteriaceae*, is the causative agent of various types of wound infections. We report a high-quality draft genome sequence of *S. marcescens* strain VGH107, which was isolated from a patient with an infection from a snakebite wound.

## GENOME ANNOUNCEMENT

*Serratia marcescens* is an important human pathogen and can present with a wide range of clinical infections. It is not only found to cause many opportunistic infections, but it is also the causative agent of various types of wound infections (1). *S. marcescens* has been reported to cause bite wound infections, but its specific functions and contribution to the disease are still unclear (2). *S. marcescens* strain VGH107 was isolated from a deep tissue culture obtained by biopsy during the initial debridement in a patient with snakebite wound complicated by fasciitis. The identification of the organism was confirmed by PCR and sequencing of the 16S rRNA genes. The genomic composition of the organism could provide valuable insights into its pathogenicity and virulence in human soft tissue infection.

The VGH107 strain was sequenced by Illumina MiSeq personal sequencer at ~300-fold coverage (1,527,281,950 bp, 151-bp paired-end reads, 240-bp insert) (3). SOAP*denovo* was first used to assemble short reads into contigs using multiple *k*-mers, where *k* = 55, 59,…, 95 (4). Note that these *k*-mers imply the minimum overlap lengths between short reads. The usage of multiple *k*-mers aims to adapt to coverage variance across the entire genome, as a small *k*-mer is good for assembly in low-coverage regions, while a larger *k*-mer is better for high-coverage regions. Subsequently, contigs assembled from different *k*-mers (average N_50_, 24,267 bp) were merged into larger metacontigs by an overlap-graph assembly called AMOS (with N_50_ improved to 220,705 bp) (5). Finally, the paired-end reads were used to link metacontigs into larger units called scaffolds by SOAP*denovo*. The final assembled genome consists of 50 large contigs/scaffolds with a total size of 5,519,754 bp, a maximum contig size of 1,005,446 bp, and N_50_ of 290,775 bp. The G+C content was 59.46%, as calculated by an in-house shell script.

Automatic annotation of the genome was performed using the NCBI Prokaryotic Genomes Automatic Annotation Pipeline (PGAAP), which utilizes GeneMark, Glimmer, and Prodigal searches. A total of 5,176 coding DNA sequences occupy 86.89% of the VGH107 draft genome. We found 57 genes related to antibiotic resistance and 91 genes related to virulence. Nontranslated genes were predicted using tRNAscan-SE (6), RNAmmer (7), and Rfam (8), which identified 86 tRNAs and 3 rRNAs.

The information provided in the genome sequence of *S. marcescens* strain VGH107 will enable further studies to understand the transmission, fitness, and pathogenesis of this zoonotic pathogen.

### Nucleotide sequence accession numbers.

This Whole-Genome Shotgun project has been deposited at DDBJ/EMBL/GenBank under the accession no. AORJ00000000. The version described in this paper is the first version, accession no. AORJ01000000.
